# Seroprevalence and Risk Factors Associated to *Mycobacterium bovis* in Wild Artiodactyl Species from Southern Spain, 2006–2010

**DOI:** 10.1371/journal.pone.0034908

**Published:** 2012-04-16

**Authors:** Ignacio García-Bocanegra, Bernat Pérez de Val, Antonio Arenas-Montes, Jorge Paniagua, Mariana Boadella, Christian Gortázar, Antonio Arenas

**Affiliations:** 1 Departamento de Sanidad Animal, Facultad de Veterinaria, Universidad de Córdoba-Agrifood Excellence International Campus (ceiA3), Córdoba, Spain; 2 Centre de Recerca en Sanitat Animal (CReSA), UAB-IRTA, Barcelona, Spain; 3 Instituto de Investigación en Recursos Cinegéticos IREC (CSIC–UCLM–JCCM),Ciudad Real, Spain; Statens Serum Institute, Denmark

## Abstract

The control of bovine tuberculosis (bTB) is at a critical point in the last stage of eradication in livestock. Wildlife species recently have emerged infected with TB in Europe, particularly ungulates in the Iberian Peninsula. Epidemiological information regarding TB in wild ungulates including affected species, prevalence, associated risk factors and appropriate diagnostic methods need to be obtained in these countries.

A cross-sectional study was carried out on wild artiodactyl species, including Eurasian wild boar (*Sus scrofa*) red deer (*Cervus elaphus*), roe deer (*Capraelus capraelus*), fallow deer (*Dama dama*), Spanish ibex (*Capra pyrenaica hispanica*) and mouflon (*Ovis musimon*), in Spain to assess the seroprevalence against *Mycobacterium bovis* or cross-reacting members of the *Mycobcaterium tuberculosis complex* (MTBC), and to provide information on associated risk factors. Previously, two in-house indirect enzyme linked immunosorbent assays (bPPD-ELISA and MPB83-ELISA) were developed using known TB status sera. Positive reference sera were selected from infected animals confirmed by culture. The *M. bovis* isolates belonged to spoligotypes SB0121, SB0120, SB0295, SB0265 and SB0134. Two hundred and two out of 1367 (7.5%; 95% CI: 6.1–8.9) animals presented antibodies against *M. bovis* by both bPPD-ELISA and MPB83-ELISA. Significantly higher TB seroprevalence was observed in wild boar compared to the other species analyzed. Interestingly, seropositivity against *M. bovis* was not found in any out of 460 Spanish ibex analyzed. The logistic regression model for wild boar indicated that the seropositivity to *M. bovis* was associated with age, location and year of sampling, while the only risk factor associated with *M. bovis* seroprevalence in red deer and fallow deer was the age. The seroprevalence observed indicates a widespread exposure to MTBC in several wild artiodactyl species in southern Spain, which may have important implications not only for conservation but also for animal and public health.

## Introduction

Tuberculosis (TB) caused by *Mycobacterium bovis*, a member of the *Mycobacterium tuberculosis complex* (MTBC), is an infectious disease worldwide extended in a large rank of hosts including humans, livestock and wildlife [1]. Because of its zoonotic nature and the high economic impact in livestock production and in animal health policies, the eradication of bovine TB (bTB) has been a major concern of public health authorities during the last three decades. In Spain, eradication programs are mainly based on intradermal tuberculin (IDT) and interferon-γ (IFN-γ) tests and culling of reactor animals [2]. Even though the application of these programs reduced bTB prevalence from 11.1% in 1986 to 1.6% by the end of 2009 with an estimated cost of €34.7 m, bTB eradication has not yet been achieved and prevalence has reached an asymptote in the lasts ten years [2].

In Spain, the control of the disease in wildlife is a critical point in the last stages of the eradication programs of bTB [3]. It is well acknowledged that the reduction in bTB prevalence in cattle is less effective in areas where cattle are extensively managed, sharing habitat with wildlife [3]. In addition, the artificial management of large game species for hunting (e.g. feeding and fencing) has significantly increased in different regions of central and southern Spain during the last decades [4]. Intensive management measures in hunting areas influence not only the population dynamics but also the behavior of the animals, promoting their aggregation, facilitating contact between individuals and favouring, therefore, the transmission of diseases among wildlife and livestock species [5]. In this sense, epidemiological, pathological and microbiological evidence strongly suggests that wild ungulates, predominantly wild boar (*Sus scrofa*) and red deer (*Cervus elaphus*), act as true TB wildlife reservoirs in the Mediterranean ecosystem [6]–[10].

Post-mortem diagnosis of TB in hunter-harvested wild ungulates is usually based on direct methods, including TB-like lesion recording, histopathological analyses and mycobacterial culture [11]. Ante-mortem diagnosis of MTBC infection in living wild ungulates can be done by indirect methods such as skin-testing and detection of specific serum antibodies [12]. Serological tests are easy, fast and inexpensive, and thus eventually more suitable than other post-mortem techniques. During the last decades, efforts to improve serological diagnosis of TB have led to the development of new serological tests. Even though *M. bovis* purified protein derivative (bPPD) is the most frequently antigen used for serological diagnosis of TB, different methods using other antigenic proteins, such as MPB83, MPB70, ESAT-6 and CFP10, and combinations of them, have been recently developed. In this sense, multiantigen print immunoassay (MAPIA) and lateral-flow-based rapid test (RT) have been shown as useful diagnosis tools in multiple host species [13]. An indirect bPPD-ELISA test has also been recently developed to detect antibodies against TB in wild boar [14], [15]. The results showed high accuracy, supporting the use of the ELISA test as complementary technique for the diagnosis of TB in wild boar. The serodominant protein MPB83 has been recently evaluated successfully yielding high sensitivity in serological assays performed in experimentally infected cattle [16] and goats [17].

The aims of the present study were (1) to analyze seroprevalence against *M. bovis* in wild artiodactyls from southern Spain and (2) to provide information on the risk factors associated with this infection. To achieve this goal, two in-house ELISAs (bPPD-ELISA and MPB83-ELISA) were developed to detect antibodies against *M. bovis*. The ELISAs were validated with sera obtained from wild ungulate species of known microbiological status (positive controls) and sera from wild ungulate species from TB-free areas (negative controls).

## Results

**Figure 1:**
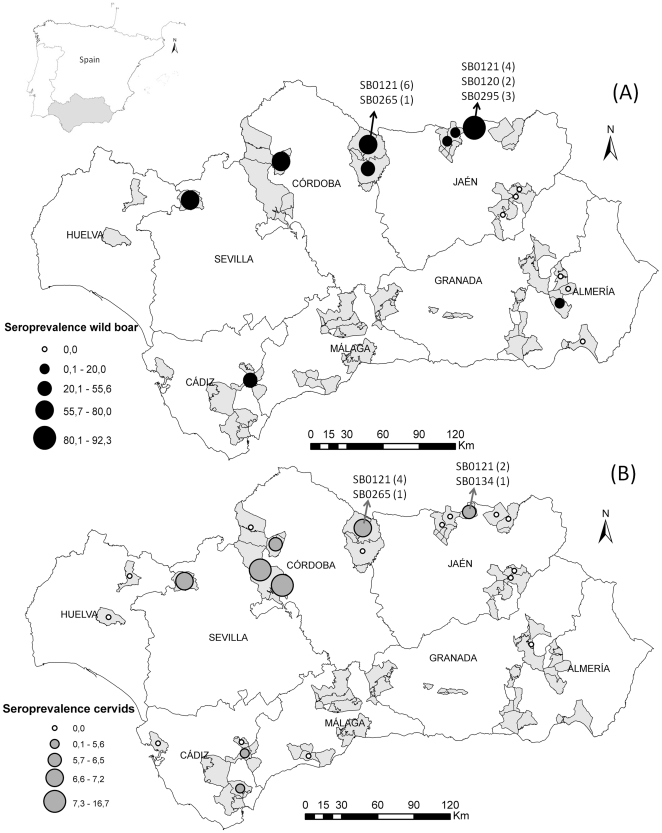
Map showing the location of Andalusia (southern Spain) and the provinces in which it is divided. Spatial distribution of wild boar (A) and cervids (red deer and fallor deer) (B) sampled in Andalusia (southern Spain), 2006–2010. Grey areas indicate the municipalities in which samples from wild artiodactyls were collected during the study period. Dot sizes are proportional to the seroprevalences against bTB for sampling areas. Spatial distribution of the different spoligotypes isolates and the number of positive animals for each spoligotype (in brackets) are included.

A total of 102 out of 1367 sera analyzed (7.5%; 95% CI: 6.1–8.9) were considered positive to TB antibodies (Table 1). Ninety-five out of the 102 positive sera were positive by both bPPD-ELISA and MPB83-ELISA. Besides, seven sera were negative by bPPD-ELISA but showed positive results by both MPB83 ELISA and mycobacterial isolation, and were therefore considered positive samples. Seventeen out of the 85 positive samples by both ELISAs also showed positive results by mycobacterial isolation. Fifty-one samples not analyzed by mycobacterial isolation, showed negative results using bPPD-ELISA but positive results using MPB83-ELISA, while 13 samples were positive by bPPD-ELISA but negative by MPB83-ELISA. The *M. bovis* isolates were confirmed as spoligotypes SB0121 (10 wild boar, 4 red deer and 2 fallow deer), SB0120 (2 wild boar and 1 red deer), SB0295 (3 wild boar), SB0265 (1 wild boar) and SB0134 (1 red deer) (Figure 1).

**Table 1:** Seroprevalence of antibodies against *M. bovis* in wild artiodactyl species from Andalusia (southern Spain) using two in-house enzyme linked assays (bPPD-ELISA and MPB83-ELISA) and mycobacterial isolation.

	bPPD-ELISA		MPB83-ELISA		bPPD-ELISA/MPB83-ELISA			*M. bovis* isolation	
Species	No. Sera	% Positive	No. Sera	% Positive	No. Sera	% Positive	95% CI	No. Samples	% Positive
**Wild boar**	130	54.6	130	67.7	130	52.3	(43.8–60.8)	18	88.9
**Red deer**	530	4.3	484	9.5	530	4.0	(3.4–5.6)	6	100.0
**Fallow deer**	128	7.0	108	13.9	128	7.0	(2.6–11.4)	2	100.0
**Roe deer**	54	3.7	59	1.9	54	1.9	(0.0–5.6)		
**Mouflon**	65	6.2	59	5.1	65	4.6	(0.0–9.7)		
**Spanish ibex**	460	0.0	312	0.0	460	0.0	(0.0–0.3)		
**Total**	1367	8.0	1147	13.3	1367	7.5	(6.2–8.8)	26	92.3

Seroprevalence among age classes, sexes, locations, and years of sampling in the different analyzed species are shown in Table 2. Prevalence of antibodies against *M. bovis* ranged from 52.3% in wild boar to 0.0% in Spanish ibex. Significantly higher TB seroprevalence was observed in wild boar compared to the other species analyzed (*P*<0.001) and in fallow deer compared to Spanish ibex (*P* = 0.016). The logistic regression model indicated that seropositivity to *M. bovis* in wild boar was associated with age class, location and year of sampling, while the only risk factor associated with *M. bovis* seroprevalence in both red deer and fallow deer was the age class (Table 3). Adult wild boar, red deer and fallow deer had significantly higher seroprevalence compared to sub-adults and juveniles. Statistically significant differences for wild boar were also found among the different sampling areas, being the highest seroprevalences recorded in Sevilla and Córdoba provinces. Significantly higher seroprevalence was also found in wild boar tested in 2010 compared to the other years of sampling. No statistically significant differences were observed in TB seroprevalence between sexes in these species.

**Table 2:** Frequency of antibodies against *M. bovis* in wild artiodactyl species in Andalusia (southern Spain).

		Wild boar		Red deer		Fallow deer		Roe deer		Mouflon		Spanish ibex	
Category	Value	No.	No.	No.	No.	No.	No.	No.	No.	No.	No.	No.	No.
		Samples*	Positive (%)	Samples*	Positive (%)	Samples*	Positive (%)	Samples*	Positive (%)	Samples*	Positive (%)	Samples*	Positive (%)
**Age**													
	**Juveniles**	18	2(11.1)	68	3(4.4)	10	0(0.0)			3	0(0.0)	50	0(0.0)
	**Sub-adults**	41	16(39.0)	268	3(1.1)	89	5(5.3)	30	1(3.3)	37	1(2.7)	85	0(0.0)
	**Adults**	71	50(70.4)	128	15(11.7)	24	4(16.7)			20	2(10.0)	204	0(0.0)
**Sex**													
	**Female**	42	22(52.4)	225	6(2.7)	61	5(8.2)	5	0(0.0)	19	1(5.3)	185	0(0.0)
	**Male**	21	9(42.9)	231	12(5.2)	60	4(6.7)	40	1(2.5)	43	1(2.3)	134	0(0.0)
**Location**													
	**Almería**	16	1(6.3)	1	0(0.0)							19	0(0.0)
	**Cádiz**			31	0(0.0)	47	4(8.5)	53	1(1.9)	37	1(2.7)		
	**Córdoba**	47	31(66.0)	162	9(5.6)	66	4(6.1)			18	1(5.6)		
	**Granada**			4	0(0.0)							230	0(0.0)
	**Huelva**			31	0(0.0)					1	0(0.0)		
	**Jaén**	32	14(43.8)	157	3(1.9)	12	1(8.3)			4	1(25.0)	46	0(0.0)
	**Málaga**	18	9(50.0)	61	3(4.9)	3	0(0.0)			5	0(0.0)	165	0(0.0)
	**Sevilla**	17	13(76.5)	83	6(7.2)								
**Year**													
	**2006**	4	2(50.0)	40	2(5.0)	7	0(0.0)			1	0(0.0)	42	0(0.0)
	**2007**	17	6(35.3)	54	3(5.6)			7	0(0.0)			35	0(0.0)
	**2008**	38	13(34.2)	66	1(1.5)	8	0(0.0)	17	0(0.0)	6	1(16.7)	231	0(0.0)
	**2009**	46	23(50.0)	274	9(3.3)	68	1(1.5)	30	1(3.3)	36	2(5.6)	152	0(0.0)
	**2010**	22	21(95.5)	96	6(6.3)	45	8(17.8)			22	0(0.0)		
**Total**		**130**	**68(52.3)**	**530**	**21(4.0)**	**128**	**9(7.0)**	**54**	**1(1.9)**	**65**	**3(4.6)**	**460**	**0(0.0)**

* **Missing values were omitted.**

**Table 3:** Results of the logistic regression model of risk factors associated to *M. bovis* seroprevalence in wild artiodactyl species in Andalusia, southern Spain.

	Wild boar				Red deer and Fallow deer			
Variable	*β*	*P*-value	OR	95% CI	*β*	*P*-value	OR	95% CI
**Age**	1.670	>0.001	2.30	(1.53–3.49)	1.792	>0.001	5.46	(2.66–11.23)
**Province**	0.402	0.008	1.49	(1.12–2.01)	-	-	-	-
**Year**	1.082	>0.001	2.95	(1.64–5.31)	-	-	-	-

When using the known positive (n = 24) and negative (n = 56) control samples, the Se results for bPPD-ELISA and MPB83-ELISA were 72.7% and 100%, respectively, while Sp for both ELISAs was 100%. Using the reference (sera from animals with bTB status previously known) and field (sera from animals with bTB status unknown) samples, the Kappa values for agreement between bPPD-ELISA and MPB83-ELISA were 0.788 and 0.697, respectively.

## Discussion

Good Kappa values for agreement between ELISAs were obtained using both reference and field samples. Although both ELISAs showed 100% Sp, the Se obtained for bPPD-ELISA was lower (72.7%) compared to MPB83-ELISA (100%). A large number of field sera (58) showed positive results by MPB83-ELISA but negative by bPPD-ELISA, seven of them could be confirmed by mycobacterial culture. The lower Se of the bPPD-ELISA or the early detection of antibodies using the MPB83-ELISA, could explain the differences between tests [16], [17]. Similar results of accuracy were previously reported using a bPPD-ELISA in wild boar [14], [15] and cattle [18]. However, a close comparison among studies is difficult to interpret because of the different cut-off values, antigens used, type of samples, infection status or species analyzed. Even though additional studies, including a higher number of reference sera, are required to evaluate the accuracy serological tests, the results suggest that the ELISAs evaluated may be suitable for surveillance programs in wild ungulate populations.

Our results confirm the current circulation of the same *M. bovis* spoligotypes among wildlife and domestic species in southern Spain. SB0121 was the most often spoligotype isolated (67%), which is in accordance with the previously reported in both wildlife and livestock in the Iberian Peninsula [6], [19], [20]. Statistically significant differences (*P*<0.05, Chi square test) in the frequency of spoligotypes isolated were observed between the present study and those previously published [19], [20]. The number of samples analyzed and the species included in the different studies are possible factors implicated in these differences. SB0120 and SB0134 have also been frequently isolated in Spain, France and Italy [21]–[23]. The geographical nearness and trade relationship could explain the similarities among countries [20].

The frequency of bTB antibodies ranged between 8.0% and 13.3% using the bPPD-ELISA and MPB83-ELISA, respectively. Even though TB seropositivity (7.5%) was determined only from samples positive by both ELISAs and by *M. bovis* isolation and, therefore, the seroprevalence could have been underestimated, the results obtained confirm that TB infection is common in some wild artiodactyl populations in southern Spain [9], [24], [25]. The high seropositivity detected in wild boar indicates widespread TB circulation in this species in the study area (Figure 1). Although high prevalence levels have been observed in wild boar in other European countries [26], the seroprevalence obtained in the present study was significantly higher (*P*<0.05, Chi square test) to those reported in countries as France, Italy and Portugal [26]. However, our results are of the same magnitude as previously obtained in Spain based on direct and indirect methods of diagnosis [9], [24], [27], and statistically significant differences in the seroprevalence to bTB between the present study and those carried out in central and southern Spain were not observed (*P*>0.05, Chi square test). High TB prevalence and increased densities in the lasts decades [3], indicate that wild boar can act as natural wildlife reservoir of TB in Spanish Mediterranean ecosystems [10]. Therefore, control measures in this species are required in order to optimize the eradication programs in Andalusia, where the incidence of bTB in cattle has increased from 5.3% in 2005 to 8.9% in 2009 [2]. Six out of 15 hunting areas analyzed presented seroprevalence in wild boar higher than 50%. The herd prevalence of bTB in cattle in these areas ranged between 3.0 and 47.6% in 2009 [2].

High differences in the prevalence of antibodies against MTBC have been reported in red deer (ranging between 0% and 50%) and fallow deer (ranging between 3% and 27%) among European countries, with the highest levels usually obtained in Spain [12], [26]. Our results were similar to those found in red deer in southwestern Spain (5.1%) [28], but lower than the prevalence levels for red deer (27%) and fallow deer (18–27%) previously obtained in southern and central Spain using direct diagnosis methods [12], [24], [25]. In this sense, the seroprevalence obtained in red deer and fallow deer in the present study was significantly lower (*P*<0.05, Chi square test) to those reported in these areas [12], [24], [25]. The lower Se of the ELISA test in these species, the density of animals or management and environmental factors in the study area may be implicated in these differences.

One additional point to consider is the possible interference of ELISA results by cross reaction of environmental mycobacteria or of mycobacteria belonging to the *M. avium* complex (MAC). Infections with environmental mycobacteria locally occur in wild ungulates from southern Spain, but at lower prevalence than MTBC infections [29]. Regarding MAC, infections with *M. avium paratuberculosis* are not common in Iberian red deer [30]. Therefore, seropositivity observed in this study is most likely due to contact with MTBC rather than other mycobacteria.

Seropositive mouflon were detected in three different regions, which suggest circulation of *M. bovis* in this species in the study area. Susceptibility to *M. bovis* infection was previously shown in domestic sheep from areas with high environmental contamination [31]. The isolation of *M. bovis* in both domestic sheep and mouflon has been recently reported in Spain [20], [32]. Additional studies are needed to evaluate the role of this species in the epidemiology of TB in Spain.

Although caprine tuberculosis caused by *M. bovis* has been widely studied in domestic goats due to their high susceptibility to infection [33], little is known about TB in wild *Caprinae*. In the present study, no seropositivity against TB was detected in the 460 Spanish ibex analyzed. In the same way, Cubero et al. (2002) [34] found only 0.2% (1/450) prevalence in Spanish ibex from southern Spain and no MTBC infection was detected in Catalonia (north-eastern Spain) among 205 ibexes from an area that sustained wild boar with a high (46%) prevalence [35]. The results might be related to natural resistance to TB in Spanish ibex, but also to the geographical distribution, behaviour, population density or management factors in this species.

TB in roe deer has been reported only sporadically in European countries, with prevalences ranging between 0% and 3% [26], [36]. Our results were in keeping with these findings, and suggest that roe deer are not a significant MTBC reservoir for other wildlife or livestock.

The higher seroprevalence in adult wild boar, red deer and fallow deer, likely reflects their greater probability of exposure and the lifelong persistence of *M. bovis* antibodies. The results were in agreement with those previously reported [10], [26], [37], [38]. Statistically significant differences in seroprevalence between sexes were not observed. In this sense, significantly higher prevalence in males was previously found in red deer [37] and white-tailed deer (*Odoicoleus virginianus*) [38]. In contrast, Santos et al. (2010) [11] reported higher prevalence in female wild boar associated to the higher gregarious behaviour of the female compared to male.

The risk of being a seropositive wild boar significantly increased in the provinces of Córdoba and Seville, which is in accordance with the higher geographical distribution of bTB in cattle [39]. Host density, management and environmental factors are possibly implicated in the spatial distribution of TB in southern Spain [28]. The highest seroprevalence found in wild boar in 2010 could indicate an increasing trend in the prevalence of contact with MTBC in this species in the study area. TB prevalence has increased in wild boar from central and south-western Spain during the last two decades [7], [8], [24], and TB is suggested to be emerging in several regions of Spain [38].

In conclusion, the results obtained using reference sera showed that the ELISA tests are potentially useful for the diagnosis and large scale monitoring of TB infection in wild artiodactyls [27], [40]. The use of ELISAs as large-scale screening tests in wildlife may be highly recommended for assessing the prevalence, mechanisms of transmission and to implement control measures. This simple and inexpensive monitoring tool could contribute to screen wild artiodactyls in other regions, notably in European countries with a recent history of wildlife TB. The identification of effective tools for TB diagnosis in wildlife is crucial for the success of the eradication programs in livestock. The high TB seroprevalence found indicates that *M. bovis* is widespread among wild artiodactyl populations in Andalusia, especially in the wild boar. The results suggest that different species could act in the maintenance and inter-specific transmission of TB in the study area, and should, therefore, be included in eradication programs in Spain.

## Materials and Methods

### Ethics statement

This study did not involve purposeful killing of animals. Samples from dead animals were collected from individuals legally hunted during the hunting seasons. No ethical approval was deemed necessary. Blood samples from captive Spanish ibexes were collected in compliance with the Ethical Principles in Animal Research in the Wildlife Breeding Centers, which depend on the Autonomous Government of Andalusia. The collection of this material did not require the approval of an ethics committee because are considered among routine procedures and the samples were collected and stored before the design of this study. Thus, protocols, amendments and other resources were done according to the guidelines approved by the Autonomous government of Andalusia.

### Field sampling

A total of 1367 samples from wild ungulates were collected in 46 hunting estates from Andalusia, (36°N to 38°60′N, 1°75′W to 7°25′W), southern Spain, between 2006 and 2010 (Figure 1). Blood or pleural fluids from hunted free-ranging red deer (n = 530), fallow deer (n = 128), roe deer (n = 54), mouflon (n = 65), Spanish ibex (n = 88) and wild boar (n = 130) were obtained from heart or thoracic cavity during the hunting season (October to March). Blood samples (n = 372) from captive Spanish ibexes were collected from the three different Breeding Centres of Andalusia, where the animals were maintained in small fenced areas. Samples were collected into sterile tubes without anticoagulant and centrifuged at 400 *g* for 15 minutes. Serum or fluid exudate samples were stored at −20°C until analysis.

Age was determined by tooth examination in wild boar, red deer, fallow deer and mouflon, and by horn segment counts in Spanish ibex [41]. The animals were classified into three age groups: yearlings (<2 year old), juveniles (between 2 and 4 year old) and adults (>4 year old).

### Control sera

A total of 26 animals (18 wild boar, six red deer and two fallow deer) which presented gross lesions compatible with bTB were tested by mycobacterial isolation. Selected animals were sampled in two different TB endemic areas of the Provinces of Córdoba and Jaén (northern Andalusia) (Figure 1) during the study period. Post-mortem examination of these animals revealed multifocal to coalescing, granulomatous, caseous and calcified nodules of different diameters in lung, and submandibular, tracheobronchial, mediastinal and mesenteric lymph nodes. TB-like lesions were collected in sterile containers and stored at −80°C until processing. A pool of sample homogenates was subjected to specific mycobacterial culture using standard procedures. The homogenates, previously decontaminated with 0.35% w/v hexadecylpyridinium chloride for 30 min, were cultured on Löwenstein-Jensen with pyruvate and Coletsos solid selective media (Biomérieux, Madrid, Spain). Identification of MTBC from suspected colonies was performed using a multiplex PCR amplification of the fragments coding for rRNA 16 S and MPB70 protein [42]. PCR was only performed on culture-positive animals. *M. bovis* spoligotypes were identified using the standardized membrane with 43 spacers as previously described [43]. Twenty-four out of 26 animals showed positive results by mycobacterial culture. Serum from these naturally infected animals (16 wild boar, six red deer and two fallow deer) were included as positive controls. Because the positive and negative animals by mycobacterial isolation were sampled during the serosurvey in the study area, sera from these individuals were also included to determine the seroprevalence against *M. bovis*.

The negative reference sera (n = 56) used in the study were obtained from 15 red deer, 15 fallow deer, 11 Spanish ibex and 15 wild boar from TB-free areas (areas where no *M. bovis* has been isolated during the last decade) in different regions of Spain. Negative reference sera were collected in previous studies by Dr. Gortázar [27], [30], [44]. The selected animals showed negative results to TB by both histopathology and mycobacterial isolation.

### Serological analyses

Serum samples were tested for the presence of antibodies against *M. bovis* by using two in-house enzyme linked assays (ELISA) using the protocol previously described [17]. All sera (n = 1367) were analyzed by bPPD-ELISA. Dilutions of bPPD (CZ Veterinaria, Porriño, Spain) in carbonate/bicarbonate buffer at a final concentration of 2 µg/ml were coated in Nunc-Multisorp™ 96-well plates (Nunc, Roskilde, Denmark) and incubated overnight at 4°C. After blockade of 45 min at 37°C with PBS containing 0.05% Tween 20 (PBS-T20) with 0.5% casein, sera were diluted 1/200 in PBS-T20 with 1% casein and subsequently added in duplicate and incubated 1 h at 37°C. After washing, a combination of Protein A and Protein G conjugated with peroxidase (Sigma-Aldrich, Steinheim, Germany) was added at a final concentration of 50 ng/ml and 100 ng/ml respectively.

In addition, a total of 1147 sera, including all positive bPPD-ELISA and 1038 negative bPPD-ELISA sera were tested by MPB83-ELISA. Unfortunately, 220 of the samples seronegative to bPPD-ELISA could not be tested using MPB83-ELISA due to limited sample volume. The antigen MPB83 (Lionex, Braunschweig, Germany) was diluted in carbonate/bicarbonate at a final concentration of 0.5 µg/ml and was coated in Nunc-Maxisorp™ 96-well plates (Nunc, Roskilde, Denmark). Antigen-coated plates were incubated overnight at 4°C. The plate blockade as well as sera and conjugated-proteins dilutions were performed as described above for bPPD-ELISA.

The reaction was revealed with soluble TMB (Sigma-Aldrich, Steinheim, Germany) and stopped 20 minutes later with 0.5 M H_2_SO_4_. The Optical Density at 450 nm (OD_450_) was then read in a plate-spectrophotometer. For each sample, the ΔOD_450_ was calculated as sample OD_450_ minus background OD_450_ (unspecific absorbance in wells where antigen had not been added). A sample was classified as positive when the ΔOD_450_ was higher than the cut-off point, calculated as the mean of background OD_450_+3×standard deviation. In the present study, positive samples to one of the ELISAs were considered undetermined samples, and were included as negative sera to determine the seroprevalence to bTB. Therefore, a serum sample was considered seropositive to *M. bovis* when showed positive results to both ELISAs or presented lesions that were culture-positive to *M. bovis*.

### Statistical analyses

Relative sensitivity (Se) and specificity (Sp) values were calculated for bPPD-ELISA and MPB83-ELISA from the reference sera. The optimal cut-off that resulted in the maximum sum of the Se and Sp values were determined by receiver operating characteristic (ROC) analysis. The Kappa value for the agreement between ELISAs was calculated using the procedure described by Kraemer (1982) [45].

Differences in ELISA performance between species (wild boar, red deer, fallow deer, mouflon and Spanish ibex) were tested using the Post-hoc Tukey test. Due to the absence of seropositivity in Spanish ibex or to the limited number of roe deer and mouflon tested, the associated risk factors were not analyzed for these species. In order to avoid potential bias associated to co-linearity, wild boar and cervids (red deer and fallow deer) were tested separately. Associations between independent variables such as age classes (yearling, juvenile and adult), sexes (male *vs*. female), years of sampling (2006, 2007, 2008, 2009, 2010), and locations (Almería, Cádiz, Córdoba, Granada, Huelva, Jaén, Málaga, Sevilla) with serological results were analyzed using Fisher's exact test. Factors showing a *P*-value<0.25 were further scrutinized for associations using Pearson correlation coefficient (*r*) to avoid collinearity problems. Finally, a multiple logistic-regression model was performed as described by Hosmer and Lemeshow (1989) [46]. All selected variables were included in the initial model and sequentially removed, starting with those with a less significant effect. The model was re-run until all remaining variables presented statistically significant values (*P*<0.05). The statistical analyses were performed using SPSS v15.0 software (SPSS Inc., Chicago, IL, USA).

## References

[1] O'Reilly C, Daborn J (1995). The epidemiology of *Mycobacterium bovis* infections in animals and man: a review.. Tuber Lung Dis.

[2] Spanish Ministry of the Environment and Rural and Marine Affairs (MARM) (2011). Reports on Spanish sanitary programs.. http://rasve.mapa.es/Publica/Sanidad/sitnat.asp.

[3] Gortázar C, Vicente J, Boadella M, Ballesteros C, Galindo RC (2011). Progress in the control of bovine tuberculosis in Spanish wildlife.. Vet Microbiol.

[4] Acevedo P, Escudero MA, Muñoz R, Gortázar C (2006). Factors affecting wild boar abundance across an environmental gradient in Spain.. Acta Theriologica.

[5] Gortázar C, Acevedo P, Ruiz-Fons F, Vicente J (2006). Disease risks and overabundance of game species.. Eur J Wildl Res.

[6] Aranaz A, de Juan L, Montero N, Sánchez C, Galka M (2004). Bovine tuberculosis (*Mycobacterium bovis*) in wildlife in Spain.. J Clin Microbiol.

[7] Hermoso de Mendoza J, Parra A, Tato A, Alonso JM, Rey JM (2006). Bovine tuberculosis in wild boar (*Sus scrofa*), red deer (*Cervus elaphus*) and cattle (*Bos taurus*) in a Mediterranean ecosystem (1992–2004).. Prev Vet Med.

[8] Parra A, García A, Inglis NF, Tato A, Alonso JM (2006). An epidemiological evaluation of *Mycobacterium bovis* infections in wild game animals of the Spanish Mediterranean ecosystem.. Res Vet Sci.

[9] Vicente J, Höfle U, Garrido JM, Fernández de Mera IG, Juste R (2006). Wild boar and red deer display high prevalence of tuberculosis-like lesions in Spain.. Vet Res.

[10] Naranjo V, Gortazar C, Vicente J, de la Fuente J (2008). Evidence of the role of European wild boar as a reservoir of *Mycobacterium tuberculosis* complex.. Vet Microbiol.

[11] Santos N, Geraldes M, Afonso A, Almeida V, Correia-Neves M (2010). Diagnosis of Tuberculosis in the Wild Boar (*Sus scrofa*): A Comparison of Methods Applicable to Hunter-Harvested Animals.. PLoS ONE.

[12] Jaroso R, Vicente J, Martín-Hernando MP, Aranaz A, Lyashchenko K (2010). Ante-mortem testing wild fallow deer for bovine tuberculosis.. Vet Microbiol.

[13] Lyashchenko KP, Greenwald R, Esfandiari J, Chambers MA, Vicente J (2008). Animal-side serologic assay for rapid detection of *Mycobacterium bovis* infection in multiple species of free-ranging wildlife.. Vet Microbiol.

[14] Aurtenetxe O, Barral M, Vicente J, De la Fuente M, Gortazar C (2008). Development and validation of an enzyme-linked immunosorbent assay for antibodies against *Mycobacterium bovis* in european wild boar.. BMC Vet Res.

[15] Boadella M, Lyashchenko K, Greenwald R, Esfandiari J, Jaroso R (2011a). Serologic tests for detecting antibodies against *Mycobacterium bovis* and *Mycobacterium avium* sub-species *paratuberculosis* in Eurasian wild boar (*Sus scrofa scrofa*).. J Vet Diag Invest.

[16] Waters WR, Palmer MV, Thacker TC, Bannantine JP, Vordermeier HM (2006). Early antibody responses to experimental *Mycobacterium bovis* infection of cattle.. Clin Vaccine Immunol.

[17] Pérez de Val B, López-Soria S, Nofrarías M, Martín M, Vordermeier HM (2011). Experimental Model of Tuberculosis in the Domestic Goat after Endobronchial Infection with *Mycobacterium caprae*.. Clin Vaccine Immunol.

[18] Ritacco V, Lopez B, Barrera L, Nader A, Fliess E (1990). Further evaluation of an indirect enzyme-linked immunosorbent assay for the diagnosis of bovine tuberculosis.. Zentralbl Veterinarmed B.

[19] Duarte EL, Domingos M, Amado A, Botelho A (2008). Spoligotype diversity of *Mycobacterium bovis* and *Mycobacterium caprae* animal isolates.. Vet Microbiol.

[20] Rodríguez E, Sánchez LP, Pérez S, Herrera L, Jiménez MS (2009). Human tuberculosis due to *Mycobacterium bovis* and *M. caprae* in Spain, 2004–2007.. Int J Tuber Lung Dis.

[21] Haddad N, Ostyn A, Karoui C, Masselot M, Thorel MF (2001). Spoligotype diversity of *Mycobacterium bovis* strains isolated in France from 1979 to 2000.. J Clin Microbiol.

[22] Zanella G, Durand B, Hars J, Moutou F, Garin-Bastuji B (2008). *Mycobacterium bovis* in wildlife in France.. J Wildl Dis.

[23] Boniotti MB, Goria M, Loda D, Garrone A, Benedetto A (2009). Molecular typing of *Mycobacterium bovis* strains isolated in Italy from 2000 to 2006 and evaluation of variable-number-tandem-repeats for a geographic optimized genotyping.. J Clin Microbiol.

[24] Gortázar C, Torres MJ, Vicente J, Acevedo P, Reglero M (2008). Bovine tuberculosis in Doñana Biosphere Reserve: the role of wild ungulates as disease reservoirs in the last Iberian lynx strongholds.. PLoS ONE.

[25] Rodríguez S, Romero B, Bezos J, de Juan L, Alvarez J (2010). High spoligotype diversity within a *Mycobacterium bovis* population: clues to understanding the demography of the pathogen in Europe.. Vet Microbiol.

[26] Wilson G, Broughan J, Chambers M, Clifton-Hadley R, Crawshaw T (2009). Scientific review on Tuberculosis in wildlife in the EU. Technical Report submitted to EFSA.. http://www.efsa.europa.eu/en/supporting/doc/12e.pdf.

[27] Boadella M, Acevedo P, Vicente J, Mentaberre G, Balseiro A (2011b). Spatio-Temporal Trends of Iberian Wild Boar Contact 3 with Mycobacterium tuberculosis Complex Detected by ELISA.. Ecohealth.

[28] Castillo L, Fernández-Llario P, Mateos C, Carranza J, Benítez-Medina JM (2011). Management practices and their association with *Mycobacterium tuberculosis complex* prevalence in red deer populations in Southwestern Spain.. Prev Vet Med.

[29] Gortazar C, Torres MJ, Acevedo P, Aznar J, Negro JJ (2011). Fine-tuning the space, time, and host distribution of mycobacteria in wildlife.. BMC Microbiol.

[30] Carta T, Martin-Hernando MP, Boadella M, Fernández-de-Mera IG, Balseiro A (2011). No evidence that wild red deer (*Cervus elaphus*) on the Iberian Peninsula are a reservoir of *Mycobacterium avium* subspecies *paratuberculosis* infection.. Vet J.

[31] Cordes DO, Bullians JA, Lake DE, Carter ME (1981). Observations on tuberculosis caused by *Mycobacterium bovis* in sheep.. NZ Vet J.

[32] Muñoz-Mendoza M, Juan LD, Menéndez S, Ocampo A, Mourelo J (2011). Tuberculosis due to *Mycobacterium bovis* and *Mycobacterium caprae* in sheep.. Vet J.

[33] Quintas H, Reis J, Pires I, Alegría N (2010). Tuberculosis in goats.. Vet Rec.

[34] Cubero MJ, Gónzalez M, León L, Pérez JM (2002). Enfermedades infecciosas de las poblaciones de cabra montés.. Distribución, genética y estatus sanitario de las poblaciones andaluzas de cabra montés.

[35] Mentaberre G, Serrano E, Velarde R, Marco I, Lavin S (2010). Absence of TB in Iberian ibex (*Capra pyrenaica*) in a high-risk area.. Vet Rec.

[36] Balseiro A, Oleaga A, Orusa R, Robetto S, Zoppi S (2009). Tuberculosis in roe deer from Spain and Italy.. Vet Rec.

[37] Vicente J, Hofle U, Garrido JM, Fernández de Mera IG, Acevedo P (2007). Risk factors associated with the prevalence of tuberculosis-like lesions in fenced wild boar and red deer in south-central Spain.. Vet Res.

[38] O'brien DJ, Schmitt SM, Fitzgerald SD, Berry DE, Hickling GJ (2006). Managing the wildlife reservoir of *Mycobacterium bovis*: the Michigan, USA, experience.. Vet Microbiol.

[39] Allepuz A, Casal J, Napp S, Saez M, Alba A (2011). Analysis of the spatial variation of Bovine tuberculosis disease risk in Spain (2006–2009).. Prev Vet Med.

[40] Boadella M, Gortazar C, Acevedo P, Carta T, Martin-Hernando MP (2011). Six recommendations for improving monitoring of diseases shared with wildlife: examples regarding mycobacterial infections in Spain.. Eur J Wildl Res.

[41] Saenz de Buruaga M, Lucio AJ, Purroy J (1991). Reconocimiento de sexo y edad en especies cinegéticas.

[42] Wilton S, Cousins D (1992). Detection and identification of multiple mycobacterial pathogens by DNA amplification in a single tube.. PCR Methods Appl.

[43] Kamerbeek J, Schouls L, Kolk A, van Agterveld M, van Soolingen D (1997). Simultaneous detection and strain differentiation of *Mycobacterium tuberculosis* for diagnosis and epidemiology.. J Clin Microbiol.

[44] Boadella M, Barasona JA, Diaz-Sanchez S, Lyashchenko KP, Greenwald R (2012). Performance of immunochromatographic and ELISA tests for detecting fallow deer infected with *Mycobacterium bovis*.. Prev Vet Med.

[45] Kraemer HC, Kotz S, Johnson NL (1982). Kappa Coefficient..

[46] Hosmer DW, Lemeshow S (1989). Applied Logistic Regression..

